# The complexity in the implementation process of empowerment-based chronic kidney care: a case study

**DOI:** 10.1186/1472-6955-13-22

**Published:** 2014-07-30

**Authors:** Annette Nygårdh, Dan Malm, Kerstin Wikby, Gerd Ahlström

**Affiliations:** 1 School of Health Sciences, Jönköping University, P.O. Box 1026, Jönköping SE-551 11, Sweden; 2 Department of Internal Medicine, County Hospital Ryhov, Jönköping SE-55185, Sweden; 3 Linneus University, Växjö SE- 351 95, Sweden; 4 Department of Health Sciences, Faculty of Medicine, Lund University, P.O. Box 187, Lund SE-221 00, Sweden

**Keywords:** Case study, Empowerment, Healthcare professionals, Implementation process, Improvement intervention, Individualized care, Interactive research, Qualitative research, Quality of care

## Abstract

**Background:**

This study is part of an interactive improvement intervention aimed to facilitate empowerment-based chronic kidney care using data from persons with CKD and their family members. There are many challenges to implementing empowerment-based care, and it is therefore necessary to study the implementation process. The aim of this study was to generate knowledge regarding the implementation process of an improvement intervention of empowerment for those who require chronic kidney care.

**Methods:**

A prospective single qualitative case study was chosen to follow the process of the implementation over a two year period. Twelve health care professionals were selected based on their various role(s) in the implementation of the improvement intervention. Data collection comprised of digitally recorded project group meetings, field notes of the meetings, and individual interviews before and after the improvement project. These multiple data were analyzed using qualitative latent content analysis.

**Results:**

Two facilitator themes emerged: *Moving spirit* and *Encouragement*. The healthcare professionals described a willingness to individualize care and to increase their professional development in the field of chronic kidney care. The implementation process was strongly reinforced by both the researchers working interactively with the staff, and the project group. One theme emerged as a barrier: the *Limitations of the organization*. Changes in the organization hindered the implementation of the intervention throughout the study period, and the lack of interplay in the organization most impeded the process.

**Conclusions:**

The findings indicated the complexity of maintaining a sustainable and lasting implementation over a period of two years. Implementing empowerment-based care was found to be facilitated by the cooperation between all involved healthcare professionals. Furthermore, long-term improvement interventions need strong encouragement from all levels of the organization to maintain engagement, even when it is initiated by the health care professionals themselves.

## Background

**Figure 1:**
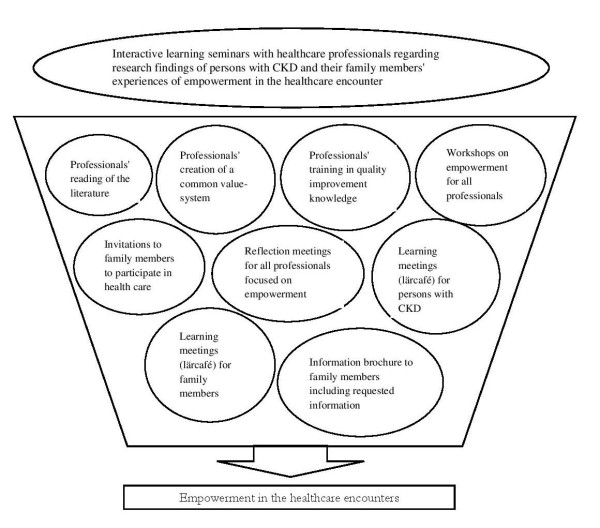
Improvement intervention to increase empowerment in healthcare encounters.

The present study is part of an interactive healthcare improvement intervention [1] using qualitative research findings [2,3] to support the intervention of empowerment-based chronic kidney care. The improvement intervention was initiated by a project group and involved the healthcare professionals at the current clinic (Figure 1). The project group initially initiated contact with the researchers. The improvement intervention applied an interactive research approach [4] and focused on partnership and learning between the researchers and healthcare professionals. The outcome of the improvement intervention has been evaluated using a quasi-experimental design and shows significant improvements in the individualization of care of the persons with chronic kidney disease (CKD). However, assessments of empowerment and coping indicate only trends of improvement [1]. In order to explain the effects of the intervention, it is of interest to understand the implementation process.

Empowerment in nursing can be described as a process that implies mobilization of the persons’ own resources to enable them to feel in control, meet their own needs, and solve their own problems [5]. There are many potential challenges in implementing empowerment-based care [6]. One of the most important difficulties is the complexity of clinical practices [7]. In addition, the importance of studying the implementation process and the context of an intervention, has been increasingly emphasized in research [8-10]. In a literature review regarding barriers for implementation, Cochrane et al. (2007) found most studies to be quantitative research [11]. To explore the context of an improvement intervention, could give valuable insights in influencing factors on outcomes [10,12,13]. Moreover, leadership and the significance for improved empowerment in health care needs to be explored [8,10,14]. The healthcare professional’s experiences of the implementation process contribute to the knowledge of what may be significant in improvement interventions in health care [9,10,13,15-17]. Furthermore, research that capture conditions at different times in the implementation process are needed to facilitate future improvement interventions in daily practice [10]. Consequently, using qualitative methods to explore the implementation process will provide valuable knowledge [9,12,15,18] and complementary to the result of the previous outcomes of empowerment-based chronic kidney care [1]. Therefore, the aim of the present study was to generate knowledge regarding the implementation process of an improvement intervention of empowerment in chronic kidney care from the health care professional perspective.

## Methods

### Design

A single naturalistic [19] qualitative case study [20] was chosen to explore the implementation process of empowerment-based chronic kidney care from the healthcare professionals’ perspective. The period of investigation prospectively was conducted over a two year period. In line with case study methodology, multiple sources of data were collected and analyzed to obtain a comprehensive picture of the implementation process of the improvement intervention [20,21].

### Participants in the case study

The study included 12 healthcare professionals from a chronic kidney care clinic, in the south of Sweden. Of these 12, seven members were from the project group who initiated the improvement intervention, and the remaining five, were staff members working with the improvement intervention outside of the project group, but still within the clinic. Age, professional background, positions, and work experience of the 12 healthcare professionals appear in Table 1.

**Table 1:** Characteristics of the healthcare professionals in the three care units (n = 58) and the study group (n = 12)

**Variables**	**Medical ward (n = 31)**	**Outpatient unit (n = 2)**	**Dialysis ward (n = 25)**	**Study group (n = 12)**
	**(n)**	**(n)**	**(n)**	**(n)**
*Sex*				
Male	2		2	
Female	29	2	23	12
*Age range*	25–55 years	48–54 years	32–63 years	33–62 years
*Profession*				
Director of the medical clinic				1
Ward manager	1		1	
Physician*				
Registered nurse	19	1	21	9**
Assistant nurse	11	1	3	2
*Education*				
Bachelor degree	7	0	1	1
Postgraduate degree	1	0	1	0
*Employed at the unit (range)*	0.5– >10 years	> 10 years	2– >10 years	4– >10 years
*Staff turnover (2009–11)*	30	1	5	
*Rotation among the care units*	1	0	0	1

*Three physicians (two men, and one woman) were organized outside the units; they worked in the outpatient unit and conducted ward rounds in the medical and dialysis wards.

**Three of the nurses dropped out of the study because of lack of time and two quit their jobs during the implementation process.

### Context

The health care professionals involved in this study were working at the chronic kidney care clinic at a county hospital in southern Sweden which provides healthcare services to approximately 125,000 inhabitants. The clinic consists of a medical ward, a dialysis ward and an outpatient unit. The three care units provide care to the same patients and have the same director. The dialysis ward and outpatient unit also have the same manager. Three physicians have medical responsibility at those three units, and they are rotated among the units involved in the improvement intervention. One of the three care units was located quite some distance from the other two units, and so the healthcare professionals did not have much opportunity to meet before the improvement intervention was instigated.

### Improvement intervention

The aim of the intervention (Figure 1) was to increase the persons with CKD, and their family member’s, empowerment by supporting and encouraging trust, learning and participation in their healthcare encounters. More details of the intervention is presented in earlier literature [1,22].

### Data collection and procedures

The multiple data collection for examining the implementation process was performed by the first author (AN).

*Project group meetings* were held once a month except during the summer vacation (July and August) period (n = 19).

*Field notes* (n = 19) included observations of the number of participants at the project group meetings and their professional positions. The field notes also included details relating to the environment of the meetings, and the positions the participants took when they seated themselves around the table. Information about someone arriving late or leaving before the end of the meeting was also recorded.

*The first interview* took place before the start of the improvement intervention process was commenced. The location was chosen by the participants, and all interviews were held in their workplace. The interviewer attempted to create a dialogue [23] that was designed to capture the details of the healthcare professional’s thoughts and expectations with regard to the improvement intervention. The interviews lasted from 23–45 minutes and started with the following open-ended questions: “Please tell me about your expectations of the improvement intervention?”, “What is the purpose of the improvement intervention?”, “How do you see your role in this intervention?”, “How do you imagine the improvement intervention will be carried out?”, and, “What are your assumptions regarding the success or failure of the improvement efforts?”. The number and content of follow-up questions depended on the interviewees’ answers.

*A second interview* was held two years later and lasted from 34–88 minutes. These interviews started with the following open-ended question: “Please tell me about your experiences regarding the work with the improvement intervention?”. Follow-up questions were asked about the process of the improvement intervention, including the participants’ experiences of the plan-do-study-act phases [24]. The final question in the first and second interviews was “Is there anything else you wish to tell me?”.

### Ethics

The participants were informed about the purpose of the study and the meaning of informed consent and confidentiality. They were given the opportunity to ask any questions before deciding whether to participate in the study. Participants were able to withdraw at any stage without any consequences, and confidentiality was guaranteed. Ethical approval for the study was obtained from the Regional Ethical Review Board at Linköping University, Sweden (Dnr: M205-08).

### Data analysis

The first author (AN) listened to the digital recordings of the project group meetings and made a transcribed synthesis of the discussions throughout the improvement intervention. Field notes were used as an additional data source to obtain a more comprehensive understanding of the project group meetings. All interviews were digitally recorded and transcribed verbatim. Inductive analysis was used, and focused on the healthcare professionals experiences throughout the implementation process. The data were analyzed using qualitative content analysis [25,26]. In the description of the analysis, the concepts commonly used in the qualitative content analysis was applied [25].

Initially, the transcribed data were imported into the qualitative data-analysis software NVIVO 8® (QRS International) [27], which was used to handle the multiple sources of data obtained throughout the whole analysis [28]. Data analysis was performed in several steps. Firstly, the data from the interviews and project group meetings were read through to obtain a comprehensive overview. Secondly, meaning units which related to the aim were identified. Next, the meaning units were labeled into codes. Following this, the codes were then abstracted and merged into categories. The categories were then abstracted and merged into sub-themes, which represented the underlying meaning of the categories. The sub-themes were then merged into three themes, which described the features for the facilitators and the barriers in the implementation process of the improvement intervention. Finally, the facilitators and barriers were sorted in three different time phases (the initiating and planning phase, the implementation phase, and the integrated implementation state). The data were reviewed, organized, and interpreted by all authors during the analysis phase, and alternative interpretations were continually discussed [25].

## Results

**Figure 2:**
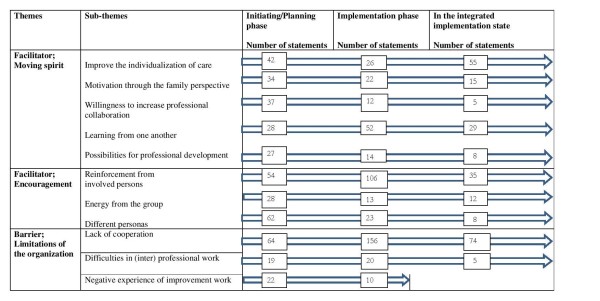
Facilitators and barrier in the implementation process.

The findings described facilitators and barriers at different phases in the implementing process of the improvement intervention of empowerment in chronic kidney care (Figure 2). As facilitators, the following two themes emerged: Moving spirit and Encouragement. One theme emerged as a barrier: Limitations of the organization.

### Facilitators and barriers in the initiating and planning phase

The theme entitled Moving spirit, was illustrated as a facilitator among the participant’s. The participants described a positive attitude to *Improve the individualization of care.* Participants expressed a willingness and readiness to implement empowerment-based care. *Motivation through the family perspective* was clearly described by all participants. Changing the perspective from a purely person-centred approach to incorporate the whole family, was a new way of thinking and one they found stimulating: “As long as you are focused on the persons with CKD and their family members . . . I believe that deep inside, most of us think that this is still very much person-centered work, and in that I think you can succeed quite well” (Interview no. 3).

The participants described a *Willingness to increase professional collaboration*. They wanted to improve the professional collaboration among, and within the different units, and hence, the “communicative walls” that existed in the current system needed to be pulled down. In addition, the participants talked about professional collaboration being essential in achieving empowerment-based care. *Learning from one another* was something that the participants thought could be accomplished through the implementation process. They emphasized the need for learning among the interprofessional project groups included in the study. The participants stated that the improvement intervention would provide *Possibilities for professional development.* They also expressed the opportunity for personal development, i.e., that the driving force in *not* remaining stationary within their profession. Rather, participants regarded the improvement intervention as an incentive for them to expand and evolve within their profession: ‘So I think when we will increase our collaboration you will exchange knowledge. So it drives me as well’ (Interview no. 2).

The second facilitator identified in the initiating and planning phase was that of Encouragement. *Reinforcement from involved persons* was achieved through external and internal sources of support. The participants described the researchers as an external facilitator who assisted with the data collection of evidence to support empowerment-based care. The participants described the importance of the improvement intervention having been approved by the director and managers at the clinic. They believed that this would provide support for the time and resources needed for the improvement intervention. The *Energy from the group* and the different personalities involved were described as essential for facilitating the implementation. This was related to their previous relationships with one another. The participants stated that they were comfortable with one another, and none of them was described as a dominant person. The participants described themselves as *Different personas* in that they probably would take different roles in the implementation process. The expressed qualities in the individuals was not related to their professional work and consisted of being organized, flexible, and taking on the role of the inspirer for keeping the implementation process ongoing: “I think it’s good when you represent different units and personalities. Then you have really all conditions to be able to get an overall picture that works, instead of working alone in each place and try to improve anyway” (Interview no. 5).

Limitations of the organization as a barrier, was described by the participants in terms of the *Lack of cooperation* among the different care units and the current lack of interplay within the organization. One of the three units was described as having an exclusive status and more resources, both in terms of staff numbers and workload. The participants referred to the importance of the improvement intervention to be in accordance with current routines to facilitate implementation. The participants described *Difficulties in (inter) professional work* in this phase*.* The members of the project group were aware of the complex issues involved in getting all their colleagues engaged in the improvement work. In the beginning they also described *Negative experiences of improvement work* as an expected barrier for the implementation of the improvement intervention. This was due to their negative experiences of earlier improvement interventions. Mostly as a result of a lack of follow-up and support: “There are many different opinions, and it can be very difficult to carry things through in this place. That was something we experienced earlier with standard-care plans and such: there are a lot of opinions about everything. So it might be what keeps us back. Well, anyway, it takes longer to get things done” (Interview no. 7).

### Facilitators and barriers in the implementation phase

The Moving spirit was described as the essence of continuous improvement in the implementation phase (Figure 2)*.* To *Improve the individualization of care* the participants had a desire to be leaders in this area. The participants stated that the implementation of the improvement intervention was feasible and not burdensome to them. Furthermore, the *Motivation through the family perspective* was described as a facilitator for a relevant and applicable implementation in their own context. The *Willingness to increase professional collaboration* was described by the participants as a collaboration opportunity to discuss mutual concerns from different angles. This was seen as necessary for the implementation of empowerment-based care. The participants also emphasized *Learning from one another* as an important facilitator for implementation of empowerment-based care. The participants’ opportunity to learn from one another was facilitated by the sharing of different perspectives with the researchers, the healthcare professionals’, and evidence on empowerment. The participants talked about an increased understanding regarding their different ways of working. The participants also spoke about how it was worthwhile learning about different methods to improve health care. The *Possibilities for professional development* was supported by the implementation of empowerment-based care. The participants described the intervention as exciting and something different from their regular work. The transformation of the concept of empowerment into the clinical context resulted in discussions that increased the participants’ professional development: “The most important thing in this project is that it's a soundly project. That it is something that will benefit staff and patients. A project you believe in, it helps to reach out to the others” (Interview no. 5).

All the participants indicated that they had received Encouragement throughout the implementation process. They described *Reinforcement from involved persons,* for example, that the researchers’ experience of health care was a source of knowledge and energy in moving forward. Inviting researchers was also described as a valuable resource to hold workshops to engage all healthcare professionals in the implementation process of empowerment-based care. In addition, external support from the local centre of improvement knowledge played an educational role. The internal reinforcement from the director of the medical clinic, and the project manager, was something that the participants described as essential, especially for the endorsement of resources and organization of the improvement intervention. The information and communication of details related to the improvement intervention was something the members of the project group declared as having a supportive function. At every workplace meeting, which were held once a month, the ongoing improvement intervention was communicated to all healthcare professionals at the three units. The *Energy from the group* assisted in facilitating the implementation phase. The participants described that the work with the improvement intervention had different impacts on their workload, and this resulted in unequal participation. However, the participants spoke about an affirmative atmosphere and a positive attitude to one another’s efforts: “The group that was formed in the beginning included people who were [hand-] picked. You knew that everyone had a genuine interest in this and wanted to do their best and then you felt supported. We knew that all of us wanted to do this and that we wanted to work for it and that we supported each other” (Interview no. 1).

The participants referred to these *Different personas* as a way to facilitate their work in spreading and implementing the activities at the three different units.

The participants expressed the Limitation of the organization as a barrier. They described a *Lack of cooperation* among the different levels of the organization, the different managers of the care units, and current routines. The project manager’s experience was that the improvement intervention did not gain approval to the same extent at all units involved in the implementation. The intervention was planned to be over a one year period. Several times in the course of implementing the improvement intervention, the director of the hospital made changes to the organization. When the improvement intervention was under way, patient records were digitized and efforts to reduce waste in health care, i.e., working in accordance with the concept of LEAN production, were introduced to all healthcare professionals. These organizational changes delayed the planned implementation. The project group described difficulties in making the same efforts in all three units. Activities that were initiated by members of the project group were sometimes difficult to achieve through lack of time. The improvement intervention was not included in their ordinary routines in daily practice, which was described by the participants as having an impact on the continuity in the implementation process. The participants’ described that putting the theoretical concept of empowerment into everyday practice, was a challenge due to the ambiguity concept: “Empowerment, it's a way of thinking which then control your actions so that but it's hard to say. How many people have done it, which has changed their thinking and working differently? It takes time” (Interview no. 6).

The participants also described *Difficulties with inter-professional work*. This was related to the different levels of engagement, qualifications, and workload of staff members. Most of the responsibility for the implementation was undertaken by the project manager. It was difficult getting physicians involved in the project. Therefore, additional lunch meetings were held to share thoughts and ideas regarding the implementation or provide information about the ongoing project. The participants in the study group stated that collaboration with the researchers sometimes hindered plans and ideas for improvement. The participants received various ideas as a result of the collaboration, but it was also necessary for them to adhere to the research process. Several discussions were held among the members of the project group as to how to put the theoretical concept of empowerment into practice. Furthermore, the participants had difficulty in maintaining the focus of the implementation over the course of time. The participants described *Negative experience of improvement work* and referred to the time spent on the project as long. They also stated that it was a challenge to maintain their level of engagement within the group. Some participants became disappointed with the project and changed from having an active to a more passive attitude. The project did not evolve as they had expected, they had thought there would be more focus on learning through information technology in order to increase empowerment for the family. Uncertainty as to the physician’s participation in meetings was also described as a disappointment in the implementation phase: “Is it possible for us to do this [reflection meeting] since it takes time? Should we include the physicians at these meetings? That would be preferable but that’s another issue. Are we to get increased collaboration? The participation of the doctors is a prerequisite, though it may be difficult to justify the physicians participating because of low staffing” (Project group meeting)*.*

### Facilitators and barriers in the integrated implementation state

Moving spirit was a facilitator for maintaining the improvement intervention. The participants emphasized the *Individualization of care* (Figure 2). They described the potential of the common value system to accomplish empowerment-based care. This facilitated the participants’ work and underlined the bottom-up approach toward improving individualized care. The *Motivation through the family perspective* was also emphasized by the participants which clarified and supported their approach to establish empowerment-based care. The *Willingness to increase professional collaboration* was responded. With the implementation of the improvement intervention, the participants described that they had less of a feeling of “us” and “them”. The *Learning from one another* resulted in an approach to work that the participants described as more responsive than had previously been the case. The *Possibilities for professional development* when establishing empowerment-based care provided the participants with a feeling of satisfaction and pride in their work: “I do think that we have started more activities than we otherwise would have done. One becomes pushed by determine what must be done together” (Interview no. 2).

Encouragement in this phase was described as the *Reinforcement from the involved persons* in the organization of the improvement intervention. The participants emphasized that reinforcement from involved persons helped sustain the improvement intervention. The two-year period of the implementation process decreased the participants’ workload and enabled them to put the improvement intervention into practice. The participants described *Energy from the group* as something that was manifested as an open-minded attitude and a readiness to entertain new approaches. This facilitated the sustainability of the improvement intervention. “The project manager presents a timeline for the improvement intervention and goes through what’s been done since the last time and what they are planning for the future . . . it’s fun to see that we really have done something . . . it becomes more real” (Project group meeting).

The limitations of the organization also described as a barrier in this phase and illustrated a *Lack of cooperation* regarding the focus on the implementation of empowerment. Participants referred to the different efforts by leaders and healthcare professionals as being an influencing factor in adopting empowerment in everyday work. The participants also spoke about the lack of time and resources after the improvement intervention: “When it [implementation] is taking place, there are a lot of discussions, but then the big discussions cease. And we are left with the difficult work situation, in which the opportunities to meet, discuss, and move forward have been lost” (Interview no. 9).

As shown in Figure 2, the findings show twice the number of statements from facilitators (747 statements), compared with the barriers (370 statements) in the implementation process. The most important facilitator in the implementation phase was *Reinforcement from involved persons*. The most commonly stated barrier in the implementation phase, was the *Lack of cooperation*.

## Discussion

To the best of our knowledge, this case study represents the first implementation process on strengthening the empowerment in healthcare encounters of persons with CKD and their family members. The main findings of facilitators and barriers in the implementation process indicate the complexity of implementation healthcare improvement.

The strongest facilitator in the *Moving spirit* was found to be the participants’ efforts to improve the individualization of care. Implementation was described as important and meaningful for the participants because the interventions were based on the feedback of persons with CKD and their family members’ experience of empowerment. This feedback was provided by the interactive researcher in interactive learning seminars with healthcare professionals. Taking the perspectives of persons with a disease into account when introducing improvement interventions in health care, have been described elsewhere [29,30]. The participants described the perspectives on empowerment of persons with CKD and their family members as assisting their own professional development. This is in accordance to earlier findings where healthcare professionals’ ongoing learning was grounded in their opportunity to reflect on clinical issues [31]. These perspective may have acted as a facilitator for transforming the research findings into practical application.

*Encouragement* from the people involved in the improvement intervention were described as a facilitator in the implementation process. The participants were involved in the clinical issues and the interactive research approach supported them in their problem solving. However, the findings also show that the interactive research approach impeded the health care professionals’ further creativity. The health care professionals need to postpone other ideas not related to the project of empowerment-based care in order to adhere to the research process. The findings showed that the nurses and assistant nurses of the project group felt they were working together to implement empowerment in healthcare encounters with persons with CKD and their family members. This result is in line with previous research findings, whereby including healthcare professionals in the improvement intervention from the beginning increases their engagement [28]. The participants described their negative experiences in earlier interventions as lack of support and follow-up from leadership. The role of leadership in this study changed in the course of the implementation from an active one, i.e., participating in every project group meeting, to the role of a discussion partner in subjects that concerned the implementation of the improvement intervention. The study found that strong support from healthcare managers, who inspire the staff in achieving a shared vision for an improvement intervention, is important to the success of the implementation [32,33]. The implementation of the improvement intervention was performed over a two year period. Due to this length of time, the participants found it as facilitating owing to the decreased workload. This finding is in line with that of previous studies, which identified the importance of an appropriate period to establish a balanced workload, while achieving behavioral changes in practice [34,35]. However, the findings also indicated that the two year period was too long for all participants to sustain their level of engagement. This findings is supported by previous research findings of patient care as having the highest priority for healthcare professionals [36]. Furthermore, the participants described feelings of disappointment in the content of the improvement intervention. Additional interventions that were out of their control were also implemented. The participants described this as having a negative impact on the improvement intervention.

Findings relating to the barriers in the implementation process pointed to *Limitations of the organization*. The participants described their awareness of this barrier in the beginning of the implementation, and their thoughts were confirmed later on in the implementation process. They described that there was a *Lack of cooperation* among the different levels of the organization and the different managers of the care units. The project manager only had authority over two units and changes were made in the organization that were out of her control and delayed the implementation process. This is in line with the results of previous studies, which identify the organization and lack of authority as a prominent barrier in the implementation of changes in healthcare [9,37]. There were also difficulties in inter-professional work. The participants referred to different skills in putting the concept of empowerment into practice. Difficulties in collective performance in inter-professional work have been detailed previously [38]. Participants also described the lack of physicians as having decreased the impact of the intervention. This is due to the fact that in the implementation process, the physicians were not participating as much as previously anticipated. The physicians were organized outside the unit, and primarily conducted ward rounds at the clinic. This is in line with previous findings that organizational constraints are a prominent barrier to physicians’ collaboration with other healthcare professionals in implementing improvement interventions [39].

The participants described their awareness of the difficulties from the start of implementing empowerment-based care. This was mostly related to the need of the improvement intervention to be in accordance to current routines. The adoption of empowerment still requires a paradigm shift [40]. Empowerment-based care requires changes to the working context [6] and close cooperation among healthcare professionals [32]. In addition to providing empowerment-based care, there is a need for comprehensive evidence to support the improvement interventions [36]. Empowerment among persons with CKD and their family members in healthcare encounters, needs inter-professional collaboration to bring about empowering encounters in health care.

Collaboration with researchers was initiated by the members of the project group. Those members described the interactive approach as having been a catalyst in the implementation process. The usefulness of an external facilitator in implementing and evaluating changes in practice, has been described elsewhere [29,41] and are related to the healthcare professionals lack of time and resources to implement research findings [36].

Efforts were made to establish the trustworthiness of the data and findings in this study [19]. Credibility was increased through the attempt to obtain a comprehensive understanding of the working context by observing ward meetings and each step in the implementation process of the improvement interventions. Interactive research has been criticized for the insufficient transferability of its results, though this is related to a specific context. This critique may be balanced by the valuable insights such research provides in the implementation process [4]. Furthermore, through an awareness of potential validity problems, the researcher was not involved in the implementation process. The use of a case study is one approach to coming close to the context in a real sense [21]. Member checking [19,21] with the participants helped increase confidence and credibility regarding interpretations of the data. Furthermore, through continual discussions over the two years within the research group during the analysis, attempts were made to increase the dependability of the results. The use of multiple sources of data about the case (implementing the improvement intervention) supported the credibility of the data through triangulation. For example, the recorded project group meetings provided some important insights that did not appear during the interviews. The participants did not describe all of the barriers in the interviews, but that was something that clearly emerged from the recorded project group meetings. Such procedures helped increase the trustworthiness in the findings of this study [19,21]. By providing a rich description of the context, this case study allows the transferability of the findings to be determined [21].

## Conclusions

The findings of this study show that the participants’ strong moving spirit and encouragement was an important facilitators to accomplish empowerment-based care. A bottom-up perspective was facilitating in that respect. The limitations of the organisation were strongly related to a lack of cooperation, which constituted a barrier in the implementation process. Implementing empowerment-based care will be facilitated through the cooperation of all involved healthcare professionals. Furthermore, long-term improvement interventions, even when it is initiated by the health care professionals themselves, needs strong encouragement from all levels in the organization to maintain engagement.

## Competing interests

The authors declare that they have no competing interests.

## Authors’ contributions

AN carried out the data collection and participated in study design, analysis, and manuscript preparation. GA participated in study design, analysis, and manuscript preparation. KW and DM participated in analysis, and manuscript preparation. All authors read and approved the final manuscript.

## Pre-publication history

The pre-publication history for this paper can be accessed here:

http://www.biomedcentral.com/1472-6955/13/22/prepub

## References

[B1] Nygårdh A Malm D Wikby K Ahlström G Empowerment intervention in outpatient care of persons with chronic kidney disease pre-dialysis Nephrol Nurs J 2012 39 4 285.2 23061113

[B2] Nygardh A Malm D Wikby K Ahlstrom G The experience of empowerment in the patient-staff encounter: the patient's perspective J Clin Nurs 2012 21 5–6 897 904 22081948 10.1111/j.1365-2702.2011.03901.x

[B3] Nygardh A Wikby K Malm D Ahlstrom G Empowerment in outpatient care for patients with chronic kidney disease—from the family member's perspective BMC Nurs 2011 10 21 22035275 10.1186/1472-6955-10-21 PMC3219548

[B4] Aagaard Nielsen K Svensson L Action and interactive research: beyond practice and theory 2006 Maastricht: Shaker Publishing

[B5] Gibson CH A concept analysis of empowerment J Adv Nurs 1991 16 3 354 361 2037742 10.1111/j.1365-2648.1991.tb01660.x

[B6] Funnell MM Nwankwo R Gillard ML Anderson RM Tang TS Implementing an empowerment- based diabetes self-management education program Diabetes Educ 2005 31 1 53 61 15779247 10.1177/0145721704273166

[B7] Rycroft‒Malone J Implementing evidence‒based practice in the reality of clinical practice Worldviews Evid Based Nurs 2012 9 1 1 22295997 10.1111/j.1741-6787.2011.00240.x

[B8] Nielsen K Randall R Opening the black box: presenting a model for evaluating organizational-level interventions Eur J Work Organ Psy 2013 22 5 601 617

[B9] Ovretveit JC Shekelle PG Dy SM McDonald KM Hempel S Pronovost P Rubenstein L Taylor SL Foy R Wachter RM How does context affect interventions to improve patient safety? An assessment of evidence from studies of five patient safety practices and proposals for research BMJ Qual Saf 2011 20 7 604 610 10.1136/bmjqs.2010.047035 21493589

[B10] Øvretveit J Understanding the conditions for improvement: research to discover which context influences affect improvement success BMJ Qual Saf 2011 20 Suppl 1 i18 i23 10.1136/bmjqs.2010.045955 PMC3066695 21450764

[B11] Cochrane LJ Olson CA Murray S Dupuis M Tooman T Hayes S Gaps between knowing and doing: understanding and assessing the barriers to optimal health care J Contin Educ Health Prof 2007 27 2 94 102 17576625 10.1002/chp.106

[B12] Hulscher MEJL Laurant MGH Grol RPTM Process evaluation on quality improvement interventions Qual Saf Health Care 2003 12 1 40 46 12571344 10.1136/qhc.12.1.40 PMC1743654

[B13] de Vos ML van der Veer SN Graafmans WC de Keizer NF Jager KJ Westert GP van der Voort PH Implementing quality indicators in intensive care units: exploring barriers to and facilitators of behaviour change Implement Sci 2010 5 52 20594312 10.1186/1748-5908-5-52 PMC2907303

[B14] Dixon-Woods M McNicol S Martin G Ten challenges in improving quality in healthcare: lessons from the Health Foundation’s programme evaluations and relevant literature BMJ Qual Saf 2012 21 10 876 884 10.1136/bmjqs-2011-000760 PMC3461644 22543475

[B15] Greenhalgh T Robert G Macfarlane F Bate P Kyriakidou O Diffusion of innovations in service organizations: systematic review and recommendations Milbank Q 2004 82 4 581 629 15595944 10.1111/j.0887-378X.2004.00325.x PMC2690184

[B16] Zapka JG Lemon SC Interventions for patients, providers, and health care organizations Cancer 2004 101 5 Suppl 1165 1187 15329892 10.1002/cncr.20504

[B17] Stirman SW Kimberly J Cook N Calloway A Castro F Charns M The sustainability of new programs and innovations: a review of the empirical literature and recommendations for future research Implement Sci 2012 7 1 17 22417162 10.1186/1748-5908-7-17 PMC3317864

[B18] van der Veer SN de Vos ML Jager KJ van der Voort PH Peek N Westert GP de Keizer NF Evaluating the effectiveness of a tailored multifaceted performance feedback intervention to improve the quality of care: protocol for a cluster randomized trial in intensive care Implement Sci 2011 6 1 119 22024188 10.1186/1748-5908-6-119 PMC3217909

[B19] Lincoln YS Guba EG Naturalistic inquiry 1985 Thousand Oaks; London: Sage Publications

[B20] Stake RE The art of case study research 1995 Thousand Oaks; London: Sage Publications

[B21] Yin RK Case study research: design and methods 2009 4 Los Angeles, Calif: Sage Publications

[B22] Nygårdh A A Quality improvement project on empowerment in chronic kidney care - an interactive research approach PhD thesis 2013 Jönköping: Jönköping University, School of health Sciences

[B23] Kvale S Brinkmann S InterViews: learning the craft of qualitative research interviewing 2009 2 Los Angeles: Sage Publications

[B24] Langley GJ The improvement guide: a practical approach to enhancing organizational performance 2009 2 San Francisco: Jossey-Bass

[B25] Graneheim UH Lundman B Qualitative content analysis in nursing research: concepts, procedures and measures to achieve trustworthiness Nurse Educ Today 2004 24 2 105 112 14769454 10.1016/j.nedt.2003.10.001

[B26] Krippendorff K Content analysis: an introduction to its methodology 2004 2 London: Sage Publications

[B27] QSR International Pty. Ltd QSR NVIVO 8 2008 Thousand Oaks, [Calif.]; Melbourne: QSR International

[B28] Ireland S Kirkpatrick H Boblin S Robertson K The real world journey of implementing fall prevention best practices in three acute care hospitals: a case study Worldviews Evid Based Nurs 2013 10 2 95 103 22730957 10.1111/j.1741-6787.2012.00258.x

[B29] Glasson J Chang E Chenoweth L Hancock K Hall T Hill-Murray F Collier L Evaluation of a model of nursing care for older patients using participatory action research in an acute medical ward J Clin Nurs 2006 15 5 588 598 16629968 10.1111/j.1365-2702.2006.01371.x

[B30] Rogers EM Diffusion of innovations 2003 5 New York: Free Press

[B31] Marshall J Images of changing practice through reflective action research J Organ Change Manag 2011 24 2 244 256

[B32] Rycroft-Malone J The PARIHS framework—a framework for guiding the implementation of evidence-based practice J Nurs Care Qual 2004 19 4 297 304 15535533 10.1097/00001786-200410000-00002

[B33] Neily J Howard K Quigley P Mills PD One-year follow-up after a collaborative breakthrough series on reducing falls and fall-related injuries Jt Comm J Qual Patient Saf 2005 31 5 275 285 15960018 10.1016/s1553-7250(05)31035-x

[B34] Francke AL Smit MC De Veer AJE Mistiaen P Factors influencing the implementation of clinical guidelines for health care professionals: a systematic meta-review BMC Med Inform Decis Mak 2008 8 1 38 18789150 10.1186/1472-6947-8-38 PMC2551591

[B35] Thompson DS O’Leary K Jensen E Scott‒Findlay S O’Brien‒Pallas L Estabrooks CA The relationship between busyness and research utilization: it is about time J Clin Nurs 2008 17 4 539 548 18205684 10.1111/j.1365-2702.2007.01981.x

[B36] de Vos ML van der Veer SN Graafmans WC de Keizer NF Jager KJ Westert GP van der Voort PH Process evaluation of a tailored multifaceted feedback program to improve the quality of intensive care by using quality indicators BMJ Qual Saf 2013 22 3 233 241 10.1136/bmjqs-2012-001375 23362504

[B37] Rycroft-Malone J Evidence-informed practice: from individual to context J Nurs Manag 2008 16 4 404 408 18405256 10.1111/j.1365-2834.2008.00859.x

[B38] Kvarnström S Difficulties in collaboration: A critical incident study of interprofessional healthcare teamwork J Interprof Care 2008 22 2 191 203 18320453 10.1080/13561820701760600

[B39] Lugtenberg M Zegers-van Schaick JM Westert GP Burgers JS Why don’t physicians adhere to guideline recommendations in practice? An analysis of barriers among Dutch general practitioners Implement Sci 2009 4 54 19674440 10.1186/1748-5908-4-54 PMC2734568

[B40] Anderson RM Funnell MM Patient empowerment: myths and misconceptions Patient Educ Couns 2010 79 3 277 282 19682830 10.1016/j.pec.2009.07.025 PMC2879465

[B41] Stetler CB Legro MW Rycroft-Malone J Bowman C Curran G Guihan M Hagedorn H Pineros S Wallace CM Role of “external facilitation” in implementation of research findings: a qualitative evaluation of facilitation experiences in the Veterans Health Administration Implement Sci 2006 1 1 23 17049080 10.1186/1748-5908-1-23 PMC1635058

